# Harnessing the Social Web for Health and Wellness: Issues for Research and Knowledge Translation

**DOI:** 10.2196/jmir.2969

**Published:** 2014-02-11

**Authors:** Kendall Ho, Peter Wall Workshop Participants

**Affiliations:** ^1^Faculty of MedicineeHealth Strategy OfficeUniversity of British ColumbiaVancouver, BCCanada; ^2^see acknowledgementsVancouver, BCCanada

**Keywords:** social media, research, interdisciplinary

## Abstract

Social media is a powerful, rapid, and popular way of communication amongst people around the world. How can health professionals and patients use this strategy to achieve optimal disease management and prevention and attainment of wellness? An interdisciplinary group at University of British Columbia, supported by a grant from UBC Peter Wall Institute of Advanced Studies, conducted a research workshop in February 2013 to explore what is known and yet to be researched in using social media for nurturing the growth of virtual communities of people for health and wellness. This two and a half day workshop brought together a group of 30 multidisciplinary experts in closed discussions to reflect on five research themes in detail: (1) individual information acquisition and application, (2) community genesis and sustainability, (3) technological design issues, (4) knowledge management, dissemination, and renewal, and (5) research designs. In addition, a public forum for the general public, which attracted over 195 live participants, over 100 participants via Web casting, 1004 tweets, and 1,124,886 impressions following the #HCSMForum hash tag on Twitter, demonstrated the keen interest of the general public in this topic. Key concepts were captured in JMIR publications in this issue, and future directions, including research, knowledge translation approaches, and strategic partnerships of interdisciplinary researchers with policy makers and industries emerged from the workshop proceedings.

## Introduction

The Journal of Medical Internet Research (JMIR) has recently published a small theme issue with three papers that emerged from a workshop on social media [1-3]. Social media is a collective term that describes a rapidly developing group of powerful and ubiquitous technologies and a set of socio-technical approaches for people to connect, support, and learn from each other [4]. Social media provides a powerful means of connectivity that can join community members in active pursuit of issues of common interest [5]. Dialogues of virtual communities formed through social media can bypass traditional methods of peer review, knowledge synthesis, and validation, thereby accelerating the dissemination and democratization of information by novices and experts alike [6-8]. Concerns have been raised that the misuse of social media forums can lead to spread of inaccurate or even harmful information, and potentially misguide participants into doing more harm than good to their wellness [9,10].

We need to understand how this popular and emerging phenomenon of social media can be effectively and safely leveraged for the pursuit of wellness and health of a community and its members. This popular and emerging phenomenon of health management calls for illumination [11]. Research is needed on how the use of social media for health and wellbeing differs across different contexts (eg, different social norms, individual attitudes and practices, professional interventions, reward structures, and learning and play) [12]. Also important is examining how health information dissemination and health practices unfold and evolves fluidly with the to and fro of interaction among participants in social media communities, contrasting this with systems designed for planned application. We need to examine the operation of new designs in social media to explore and gain a greater understanding of the impact of online tools and concepts such as open source, peer production, crowdsourcing, virtual communities, participatory culture, e-learning, and game play, on the wellbeing and health of their users.

A multi-disciplinary approach would benefit this examination, studying the issue through different research traditions, including but not limited to the social sciences (sociology, anthropology, psychology, communications), knowledge science (library science, education), technologies (engineering, computer science), management science (business engineering), and health sciences (medicine, nursing, public health). Collectively, studies from different research areas, such as social informatics, the science of socio-technical systems, eHealth, e-science, e-learning, management information systems and human-computer interaction, information science, computer science, management science, sociology, psychology, and anthropology will provide a more complete picture on the topic.

The aim of our workshop, “Harnessing the Social Web: Communities for Health and Wellness” and the study was to gather an interdisciplinary group of researchers to examine the issues of using social media for supporting health and wellness.

## Scope of the Peter Wall Workshop

A group from 5 faculties at the University of British Columbia (UBC)—Medicine, Library Science, Nursing, Sauder School of Business, and Engineering—successfully obtained a competitive grant funding from the UBC Peter Wall Institute of Advanced Studies to organize a research workshop to invite experts in various disciplines who share a common interest in social media and the use of the Web for wellness and health to explore the various dynamics at play in social media that contribute to success or failure, and the related management of information, social support, decision-making, and action in support of individual and community wellness goals. The purpose of the workshop was to bring together a group of experts to pool knowledge in support of a multi-faceted understanding of social media use in health and wellness by and for individuals and communities to explore 6 research themes (see Textbox 1).

**The 6 research themes discussed at the Peter Wall workshop.**

1. *Individual information acquisition and application:* How do individuals obtain, disseminate, and trust information obtained via social media, and how do these interactions influence change in attitude, commitment, knowledge, and behavior over time? How does the public nature of this information affect willingness to contribute? How do different cultural approaches to health and wellbeing influence individual and group behaviors?
2. *Community genesis and sustainability:* What are the dynamics that lead to the successful formation, growth, and sustainability of the virtual community facilitated by social media, and what different types of roles do individuals play to animate and enliven this community (leaders, lurkers, contributors, knowledge experts, etc)?
3. *Technological design issues:* What are the design strategies used in mobile phones, tablets, or the Web that create an optimal and positive experience for participants of the social media? For example: ease of access, quality of communication, speed of connectivity and optimal decision support to encourage positive change and ethical attention to values, privacy, etc. What are the ethical considerations in design? How should such technologies be designed to preserve the privacy, anonymity, confidentiality of individuals and their contributed data?
4. *Stakeholders’ mutual influence towards wellness:* How do different stakeholders interact amongst and between themselves (eg. health professionals, health consumers) towards change, and what effective social phenomenon is at play in facilitating interaction among different groups? How has consumer knowledge shifted the authority previously held by health care providers?
5. *Knowledge management, dissemination, and renewal:* How would the information in the social media dialogues merge and contribute to evidence based information in support of local and outcome-specific contexts? How would this information be best represented and visualized such that virtual community members can understand and apply it? How can we make best use of two-way flow of information and experience from novices to experts, and from individuals to crowds and communities?
6. *Research designs*: What methodological approaches and research traditions provide the right kind of design and ethical practices for examining social media use by an ecology of patient and health care providers, and for capturing the data necessary to address the questions above?

## Workshop Program Overview

**Figure 1:**
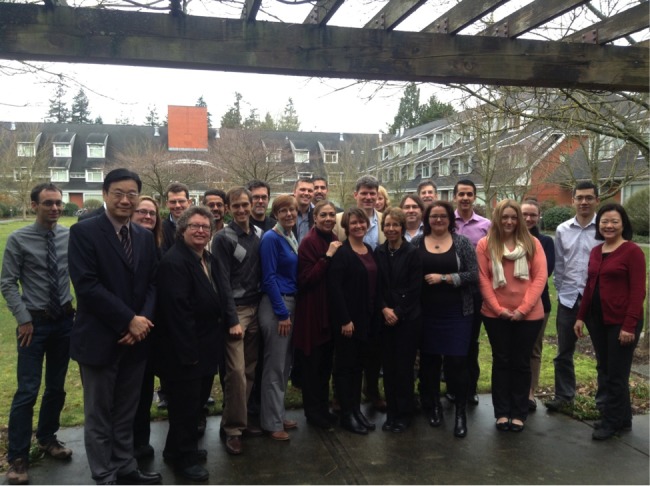
Participants of the Peter Wall Institute of Advanced Studies funded workshop "Harnessing the Social Web for Health and Wellness: Issues for Research and Knowledge Translation", in Vancouver, Canada, February 3 - 5, 2013.

We successfully invited a group of 30 multidisciplinary experts from library and information science, medicine, engineering, computer science, nursing, public health, communications, anthropology, education and sociology, health policy, and business and health economics to participate in this workshop, which took place from February 3 to 5, 2013 (Figure 1). The experts were engaged to consider the 6 research themes (Textbox 1), and produced background papers in preparation for the workshop. We also engaged graduate students from interdisciplinary studies, including the School of Population and Public Health, School of Nursing, School of Library, Archival and Information Studies, and Department of Computer Science to participate in our discussion and contributed their ideas and insights to our discussion.

During the three days of the workshop, we maximized interactions amongst participants in order to draw upon their own disciplines’ perspectives and experiences on social media for health and identify interdisciplinary opportunities and gaps needing further illumination and research. We concentrated on generating academic papers to capture the multi-dimensional ideas discussed at the workshop, resulting in 3 full papers and this editorial in the Peter Wall workshop theme issue in JMIR [1-3].

On the evening of February 4^th^, 2013**,** we conducted a public forum to present issues of using social media for wellness, inviting a few members from our workshop to give a keynote presentation and to be panellists of the forum. We invited the audience to engage in a dialogue to explore issues and ideas from their perspectives, from which we created a public forum, titled *“Social Media: The Good, the Bad and the Possible,”* which brought together the general public, digital and health care experts, dignitaries, and researchers to engage in dialogues on how social media can be used to improve their own health and wellness. The forum, which attracted an audience of over 195 live participants locally, over 100 participants via Web casting, 1004 tweets, and 1,124,886 impressions following the #HCSMForum hash tag on Twitter from BC and beyond, demonstrated the keen interest of the general public in this topic.

## Workshop Results

The success of this workshop was built on the intellectual diversity and collaboration of a group of multidisciplinary experts, enriched by the experiences of users of social media in the public. Our primary intention was to address a health audience and a social media audience of fellow researchers and academics in different disciplines who are interested in learning about and/or engaging in ways social media can be harnessed in the service of health and wellbeing. Our secondary audience included: (1) the general public to give us insights on the everyday use of social media and health practices to potentially participate with us in our research; (2) research institutions’ decision makers to raise their interest in this area of research to support further exploration of this important sociotechnical phenomenon; and (3) knowledge users wanting to leverage this medium to help improve wellness of our citizens (health policy makers, businesses and corporations, engineering/computer science enterprises, social scientists, health professionals).

The pre-workshop preparations on the 6 questions, and the input and discussions at the research workshop and the public forum, served as the basis of the 3 manuscripts associated with this workshop published in JMIR [1-3]. Their content will not be detailed in this editorial, but readers are encouraged to access these papers. The discussions in the workshop brought out several key insights which crossed disciplinary boundaries, highlighted in Textbox 2. These are essential questions and issues that need to be further investigated and addressed through interdisciplinary research.

**Key multidisciplinary insights from discussion in the Peter Wall workshop.**

- *What’s the evidence:* While there was a common notion that social media can be very helpful to support health and wellness, how do we quantify these benefits? What evidence needs to be generated to support this impression?
- *The public:* How could social media optimally and effectively support the public’s active pursuit of health and become experts of their own wellness? How could we improve the different segments of the public, including but not limited to age, language and technological literacy, types of social media used (Eg, twitter, facebook) in accessing the information and knowledge captured in the various dimensions of the social media?
- *Health professionals:* Health professionals are not embracing social media as rapidly as the public for health services or information dissemination. How could we incentivize health professionals to embrace social media, track its evolution, and work with the public to fully unleash the power of this medium to support health and wellness? Meanwhile, is there a professional obligation for health professionals on social media to respond to inquiries or address misinformation if they choose to participate in social media?
- *The information on social media:* Is the depth of the information or knowledge shared on social media appropriate for pursuit of health? How could the interactivity of the dialogues on social media be best used to support health and disease management? How to build trust and credibility to nurture the therapeutic relationships between health professionals and the patients via social media? How to increase involvement of individuals, and what types of roles are needed amongst the different participants to sustain social media discussions? Should these roles be assigned or could they be allowed to emerge spontaneously as the virtual community grows?
- *The evolution of social media:* The disruptive nature of social media to alter communal knowledge sharing is creating positive tension to stimulate changes. How can we harness this momentum of change towards empowering health consumers and health professionals alike in knowledge exchange and dissemination in health? What types of electronic tools within and outside of social media will continue to emerge in the future? How can individuals keep abreast of these changes and continue to adopt them for health and wellness?

## Next Steps

Together, the workshop and public forum shed light on the need to engage the general public, health professionals, researchers, and innovators to work together to unleash the power of social media and modern information technologies. The workshop further shed light on new and exciting ways to leverage social media in health care and to improve our health system’s ability and capacity to support each of us in wellness and sickness alike. In addition to disseminating the findings through publications, we were delighted to be engaged by the British Columbia Patient Safety and Quality Council to co-organize a follow up workshop, scheduled to occur in early 2014, to uncover the most beneficial ways of leveraging social media to promote quality and health in BC. Insights and lessons learned from this workshop will also be incorporated into the eHealth educational curriculum of medicine at UBC, and nationally through the Royal College of Physicians and Surgeons of Canada eHealth Expert Working Group and the Association of Faculties of Medicine of Canada eHealth education Committee.

We further intend to maintain connections with our workshop and public forum participants, and invite others interested in this endeavour to join and continue to explore this subject. For more information, please contact the corresponding author.
